# Recent Developments in the Application of Bio-Waste-Derived Adsorbents for the Removal of Methylene Blue from Wastewater: A Review

**DOI:** 10.3390/polym14040783

**Published:** 2022-02-17

**Authors:** Hamad Noori Hamad, Syazwani Idrus

**Affiliations:** Department of Civil Engineering, Faculty of Engineering, Universiti Putra Malaysia, Serdang 43400, Malaysia; gs59432@student.upm.edu.my

**Keywords:** methylene blue, activated carbon, agro-waste, wastewater, adsorption, cationic dyes, low-cost adsorbents, bio-waste

## Abstract

Over the last few years, various industries have released wastewater containing high concentrations of dyes straight into the ecological system, which has become a major environmental problem (i.e., soil, groundwater, surface water pollution, etc.). The rapid growth of textile industries has created an alarming situation in which further deterioration to the environment has been caused due to substances being left in treated wastewater, including dyes. The application of activated carbon has recently been demonstrated to be a highly efficient technology in terms of removing methylene blue (MB) from wastewater. Agricultural waste, as well as animal-based and wood products, are excellent sources of bio-waste for MB remediation since they are extremely efficient, have high sorption capacities, and are renewable sources. Despite the fact that commercial activated carbon is a favored adsorbent for dye elimination, its extensive application is restricted because of its comparatively high cost, which has prompted researchers to investigate alternative sources of adsorbents that are non-conventional and more economical. The goal of this review article was to critically evaluate the accessible information on the characteristics of bio-waste-derived adsorbents for MB’s removal, as well as related parameters influencing the performance of this process. The review also highlighted the processing methods developed in previous studies. Regeneration processes, economic challenges, and the valorization of post-sorption materials were also discussed. This review is beneficial in terms of understanding recent advances in the status of biowaste-derived adsorbents, highlighting the accelerating need for the development of low-cost adsorbents and functioning as a precursor for large-scale system optimization.

## 1. Introduction

The pervasiveness of pollutants in the ecosystem is often linked to population growth and anthropogenic activity [1]. Water resource contamination is an extremely contentious issue on a worldwide scale, as it has long-term or even lethal consequences for living creatures [2]. Dyes in effluents are a severe issue since they harm many sorts of life [3]. Toxicological and aesthetic issues are intertwined with regard to color dye pollution [4]. According to recent data, approximately 100 thousand commercially dyed products with a total 7 × 10^5^ tons of yearly production of dyestuff (about 10% of dyes used in industrial applications) have been released into the aquatic environment [5,6,7]. The water pollution issue was first caused by the textile industry, followed by the printing industries, as well as paper, paint, and leather production companies [8,9]. The amount of textile wastewater generated per year in the United States, United Kingdom, and China was estimated to be around 12.4, 1, and 26 million tons, respectively. This is equivalent to 1–10 million liters of textile wastewater being produced per day [10].

Over a third of the world’s renewable freshwater resources are used for industrial, residential, and agricultural purposes, and the majority of these activities pollute water with a wide range of geogenic and synthetic substances, including dyes, pesticides, fertilizers, radionuclides, and heavy metals. [11,12]. As a result, it is not surprising that water poisoning induced by a variety of human activities has created alarm regarding public health problem on a global scale. Dye-induced water pollution is one of the most serious pollutants since it alters water. Even at extremely low quantities, water retains its natural look [13,14]. These industries consume a vast proportion of the coloration and produce dye-laden effluent that is eventually released straight into the environment, posing significant environmental problems due to the dyes’ toxic and unpleasant properties [15].

MB is much more commonly used dye and is a heterocyclic molecule with the chemical formula C_16_H_18_N_3_SC_l_. Initially, it was manufactured as a synthetic aniline dye for textiles in 1876 by Heinrich Caro of Badische Aniline and Soda Fabrik. Its utility in staining and inactivating species of microbes was also revealed [16]. Additionally, it was identified in 1932 to be a cyanide and carbon monoxide antidote [17]. The ingredient is a dark green powder that causes water to turn blue at room temperature. It absorbs the most visible light at around 665 nm. MB is known to be an extensively explored dye because of its favorable and negative qualities. Its application has a wide range, with it being used in the pharmaceutical and textile industries as a coloring, as well as in the plastic, tannery, cosmetics, paper, food, and medicinal industries, and it is also used as a staining agent for the classification of microorganisms [18,19]. On the other hand, MB has garnered considerable attention due to its antagonistic nature, which has a detrimental effect on human health and the environment. This dye’s adverse effects include skin irritation, as well as mouth, throat, and stomach irritation; in addition, esophagus irritation, nausea, gastrointestinal pain, headache, diarrhea, vomiting, fever, dizziness, and high blood pressure are all common side effects of this dye [20]. The discharge of colored waste without sufficient treatment can cause severe environmental effects, including an increase in toxicity via an increase in water bodies’ chemical-oxygen demand (COD) [21]. Due to the fact that synthetic dyes in wastewater cannot be effectively decolored using currently available technologies as a result of their synthetic roots and predominantly aromatic structures, which are not biodegradable, the need to remove color from waste effluents has grown in importance. Several strategies for removing MB from waste water have been studied, including enzymatic procedures, photodegradation reactions, electrochemical extraction, membrane filtration, physical adsorption, and chemical coagulation [22,23].

Adsorption as a physico-chemical treatment has been identified as one of the most appropriate methods and has been extensively explored for MB elimination, with its total use cases more than doubling in the last decade. The adsorption approach employed a straightforward procedure with a cheap and plentiful adsorbent, and it was also capable of achieving a high removal efficiency of MB [24,25]. Additionally, adsorption prevents the formation of secondary contaminants due to the reactions of the oxidation or degradation processes of MB [26,27]. As a result, the findings have attracted the interest of numerous researchers over the last decade.

**Figure 1:**
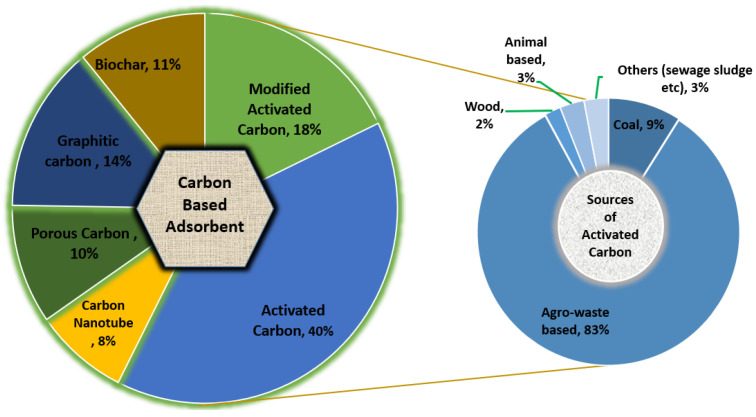
Research on carbon-based adsorbents and sources of activated carbon for methylene blue elimination from 2008 to 2021.

Most of the recent studies on adsorbent development focus on the application of carbon-based adsorbents, including magsorbents [28], nano catalyst applications [29], and the function of all types of carbon-based adsorbents [30] for MB’s removal from wastewater. To the best of our knowledge, no recent literature has addressed the removal of MB through the extensive use of bio-waste-derived adsorbents and compared the bio-waste-derived adsorbents’ characteristics as well as related parameters that influence the performance of the process. Aiming at the further evaluation of current advances and methods developed in previous studies, this review also highlights regeneration processes, economic challenges, and the valorization of post-sorption materials. This article provides new perspectives for the development of adsorbents, serving as a precursor for large-scale and low-cost adsorbent applications. Figure 1 depicts the trends in the research on the removal of MB from wastewater using carbon-based adsorbent and sources of activated carbon published between 2008 and 2021.

## 2. Carbon Structural Characteristics and Their Relationship to Adsorption Capacity

Carbon’s adsorbent quality is determined by its sorption capacity. The characteristics of the adsorbent are considered to be the most critical factors that can affect MB’s adsorption, and include the surface area, pore structure, carbon particle size, surface acidity, and functionality [31,32,33]. As illustrated in Table 1, carbon adsorbents can be classified as superior (adsorption capacity >1000 mg/g), excellent (500–1000 mg/g), moderate (100–500 mg/g), and weak (adsorption capacity 100 mg/g) based on their MB adsorption capacities. The surface area of carbon adsorbent was reported to be positively correlated with its adsorption capacity. Nonetheless, not all carbon adsorbents follow this trend, as some have low adsorption capabilities due to having excessive surface areas. The highest MB adsorption capacity, exceeding 800 mg/g, was found in adsorbents with large surface areas but small pore diameters. The MB dimensions of 0.400 × 0.793 × 1.634 nm were reported in water. In terms of facilitating MB’s diffusion via the adsorbent’s pores, the pore opening size is critical. At its maximum, carbon was found to have an adsorption capacity of 1791 mg/g, a surface area of 2138 m^2^/g, and a pore diameter of 3.33 nm [34]. Interestingly, pores with dimensions of greater than 6 nm, with total surface areas of 500 m^2^/g, were reported to have less adsorption capability than other adsorbents.

## 3. Wastewater Treatment Methods for MB’s Removal

**Figure 2:**
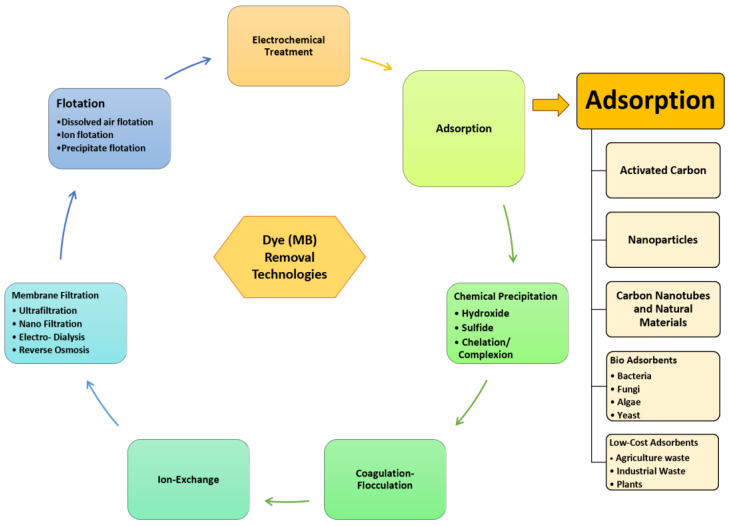
Schematic diagram of tertiary treatment for dye (MB)-removal technologies.

Dye users, industrial entities, and the government should take all appropriate steps in the treatment of dye effluents in order to improve public health and protect the environment. In general, industrial wastewater treatment technologies are divided into several stages, including pre-primary, primary, secondary, and tertiary processes [60]. The initial one is a preliminary process that is applied for the removal of contaminants (such as papers, grits, wood, plastics, cloths, etc.) with minimal effort, as well as the comminution and screening of floating, suspended particles, and oil and grease traps. The following process is the primary treatment, which includes skimming to remove frothy solids and flotation and sedimentation to remove settleable inorganic and organic impurities. Secondary wastewater treatment involves the microbial breakdown of dissolved organic and colloidal materials, which maintains the waste’s stability [61]. Biological agents are used in advanced and tertiary treatment (i.e., anoxic, aerobic and anaerobic, facultative, or a mix of these), chemical (ozonation, fenton reagents, chemical precipitation, ion exchange, photocatalysis, ultrasound, and solar-driven processes) or physical (sedimentation, membrane filtration, coagulation and flocculation, ultrafiltration, nanofiltration, adsorption, and reverse osmosis) strategies for treating effluents that are incapable of being removed during secondary treatment [62,63,64]. Likewise, during treatment of effluent-containing dye, there could be substances left in treated wastewater which require post treatment including the application of bio-waste-derived adsorbent. Previous studies reported on the disadvantages of various wastewater treatment, including lower efficiency, greater capital or operating costs, a large amount of sludge production, and high costs of maintenance, that make these technologies inappropriate for economic application [65,66]. In contrast, adsorption technology offers a wide range of techniques due to its cost efficiency, ease of operation, low energy consumption, simple set up, toughness towards harmful contaminants, capacity to eliminate all dyes, and great efficiency [67,68]. Furthermore, no harmful materials are generated as a consequence of using this treatment method. Figure 2 depicts tertiary treatment and adsorption technology as an alternative for MB’s removal from wastewater.

Current color removal treatment approaches involve chemical, physical and biological processes. There are two sorts of dye molecules: chromophores, which provide colors, and auxochromes, which not only act as a substitute for the chromophore but also increase the solubility of dye in water, thus increasing its affinity (ability to join) to fibers [69]. Chemical, physical, and biological remediations are the most often used ways for treating colored wastewater. These technologies, however, have both advantages and disadvantages. Most of these traditional procedures are inapplicable on a broad scale because of the high expense and disposal issues associated with the significant the quantity of sludge produced in the final treatment process [70].

### 3.1. Physical Techniques

Membrane filtration, reverse osmosis, electrolysis, and adsorption technology are classified as physical treatment methods. The main disadvantage of the membrane technique, in particular, is the short life due to fouling, and thus, frequent maintenance is needed. As a result, costs associated with periodic chemical cleaning and replacement have to be considered during the evaluation of its viability economically. The adsorption procedure is considered to be the most effective way for water purification among all physical treatments [71]. Adsorption is acknowledged as a potential strategy with substantial significance in the decolorization process, due to its simplicity in operation and comparably cheap application. From the point of view of its commercial scale potential, activated carbon is an extraordinary substance that is sustainable in treating polluted groundwater and industrial contaminants such as colored effluents. These natural adsorbents have been studied extensively to recover undesired hazardous chemicals at a relatively low cost from polluted water [72]. Nevertheless, the application of activated carbon is limited due to its expensive cost; thus, improvement in terms of development and regeneration is indispensable. Numerous non-traditional low-cost adsorbents have also been proposed, including zeolites, clay materials, agricultural wastes, siliceous material, and industrial waste products, in an attempt to develop more affordable and effective adsorbents [73,74].

### 3.2. Chemical Techniques

Coagulants and flocculants are the primary agents used in the treatment of dye wastewater chemically [75]. It is accomplished by adding chemicals to the influent, such as ferric ion aluminum and calcium, to produce flocs [76]. Moreover, the utilization of various chemical agents, for instance, ferric sulphate, polyaluminium chloride, and several organic synthetic polymers, in chemical treatment was previously reported [77,78]. The combination of more than one coagulant or flocculant could be applied for improving the removal rates, as suggested by Shi et al. [75]. In a nutshell, the chemical technique is generally economical and efficient, but the main disadvantage is that chemical cost is high, and prices fluctuate in the market due to the demand and manufacturing cost. Furthermore, despite its efficiency, major drawback of this technique is the formation of large sludge volume, which causes disposal issues including higher operating costs and pH dependence, thus limiting its application as a biofertilizer [79].

### 3.3. Biological Techniques

Biological treatment is the most cost-effective treatment method as compared with physical and chemical treatments. In the treatment of industrial effluents, biodegradation technologies including the use of adsorbents as alternatives for filter media to promote microbial population, have gained attention for treating bio-waste in fungal decolorization processes. Microorganisms such as algae, yeasts, fungi, and bacteria can accumulate and decompose various contaminants; however, their applications are frequently limited due to technical limits. Aerobic and anaerobic biological treatments are both possible [80]. Conversely, the main disadvantage is that it requires a large area of land and is restricted by sensitivity to diurnal change as well as chemical toxicity [73]. Furthermore, contrary findings were published in a study of existing technologies [81], which reported that the biological remediation process is incapable of achieving good color eradication while utilizing present conventional technologies. Furthermore, due to their complicated chemical structure, synthetic organic origin, and xenobiotic character, azo dyes are not easily biodegradable. Table 2 summarizes the benefits and drawbacks of different approaches for treating dye-contaminated water.

Despite significant advances in dye wastewater treatment methods, achieving commercially viable, cost-effective, and short-retention-time treatment remains a challenge. A previous study concentrated on an adsorption technique for dye treatment from wastewater [87]. This approach is capable of handling relatively high flow rates while creating high-quality effluent that does not develop hazardous chemicals such as free radicals and ozone [88]. Furthermore, it can eliminate or reduce a variety of contaminants, giving it a broader range of applications in the controlling of pollution. Adsorption is thus acknowledged as the most adaptable technique employed in less developed countries, and it is now widely used for the removal of organic pollutants from aquatic environments [89].

## 4. Adsorption

**Figure 3:**
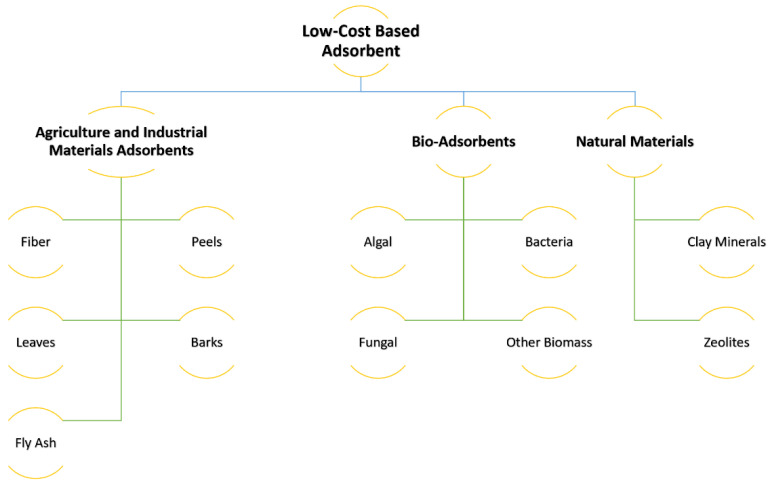
Numerous inexpensive adsorbents’ capacities for dye (MB) elimination.

The adsorption process is an efficient, affordable, and widely utilized color removal approach [90]. Biomass is commonly used as a low-cost activated carbon in wastewater remediation for the removal of impurities. Several non-conventional and cost-effective biomass-derived adsorbents have been studied in relation to the treatment of dye-containing wastewater, as shown in Figure 3. 

Environmentally friendly sorbents, which include organic waste compounds (compounds from leaves, barks, and peels) and microbial biomass (fungus bio-sorbents, green algal, and bacterial biomasses), are gaining popularity as types of commercial activated carbon (CAC). Likewise, carbon nanomaterials (graphene, carbon nanotube, and derivatives) have also been employed for decolorization [91]. Zeolite, as an inorganic adsorbent and activated carbon, can be categorized as a type of carbon compound with high oscillation and internal surfaces. Special techniques for producing them in the form of granular, powdered, and spherical activated carbons have been devised. Activated carbon is made by pyrolyzing carbon or carbon-containing plant materials such as coal, bamboo wood, charcoal, kernels, or fruit shells, for example, coconut shells [92]. Carbon can be activated by steam, carbon dioxide, or chemical means, thus making it an ideal material for dye binding. Steam activation is the most eco-friendly and cost-effective approach, whereas chemical activation leads to the highest porosity and surface area. Following the activation process, carbon can be easily rinsed and dried to eliminate the chemicals used (including acid) [92]. In terms of the sorption capacity of carbon groups, the highest theoretical adsorption capacities were recorded at 348, 527, and 394 mg/g at 25° C for Norit Darco 12–20 (DARCO). 

Charcoal-derived activated carbon was revealed to be the most superior adsorbent with an efficiency of 99.8%, and it can handle different types of dyes. Researchers discovered that MB performed better as an adsorbate as compared to Rhodamine B in wastewater [93]. At a pH of 2 and a temperature of 25 °C, the highest capabilities of microalgae and CAC in the adsorption of dye were 482.2 mg/g and 267.2 mg/g, respectively. Dye was removed at a rate of 93.6–97.7% using AC and at a rate of 94.4–99.0% with microalgae. In another investigation, CAC outperformed olive stone activated carbon in the adsorption of Remazol Red [94]. The replacement of CAC via the development of alternatives requires comprehensive research on activation methods and adsorbent characteristics. The initial dye concentration, pH, temperature, adsorbent dose and type, and contact duration are the parameters that determine the dye-adsorption ability. Effective adsorbents should have the capacity for high adsorption amounts and quick adsorption rates, be effective against a range of dyes or pollutants, and be easily regenerable and reusable to ensure efficient treatment [95]. 

Despite the good functioning of activated carbon, which has successfully removed dyes from industrial wastewater effluents, it has drawbacks such as high capital costs, high energy consumption, and sorption–desorption cycles. For color and heavy metal elimination, bio adsorbents made from bacteria or fungi are promising ecologically acceptable adsorbents [90].

## 5. Adsorption Mechanism

**Figure 4:**
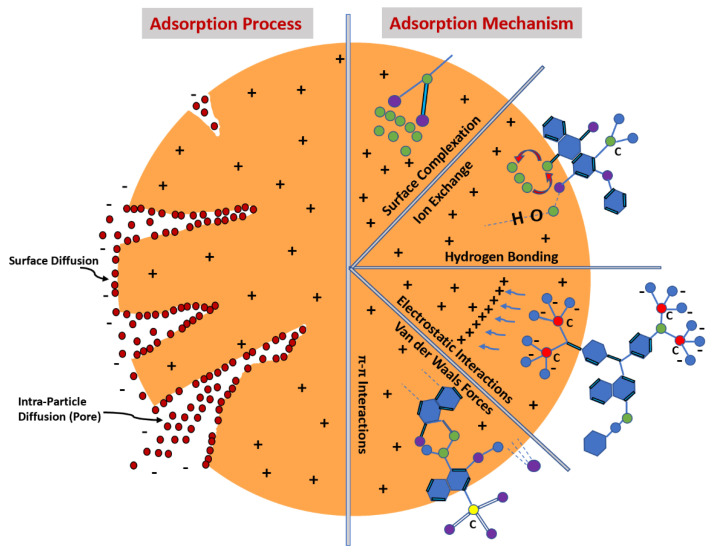
Mechanism and adsorption process for the elimination of dye (MB).

Functional groupings such as the aromatic ring, —C=O, —C—O—C-, —OH, —NH_2_, —C=S, —C=N, and —S=O on the carbon surface also play important roles in improving the adsorption capacity in terms of MB’s disconnection from water [96,97]. MB is a positively charged chemical. It has a six-carbon aromatic ring, sulfur, and nitrogen in its chemical structure. Figure 4 shows that the electron dispersion forces between the carbon surface functional groups and MB molecules induce, via electrostatic contact, hydrogen bridge generation, electron donor–acceptor relationships, and π—π forces after MB’s adsorption on carbon [57,59]. Most commonly, thermal activation involves the annealing of carbon adsorbent at high temperatures with nitrogen gas (N_2_) flowing through it. Furthermore, MB’s adsorption capacity can be maximized by increasing the carbon’s porosity and surface area. This technique is known as the addition of carboxyl group numbers (—COOH) [40,41]. Another technique to improve carbon surface functionality is to use compounds that contain the functional groups required for the chemical activation of the composites. Carbon from bio-waste is treated with propylene diamine, ethylene diamine, aniline, and ethylene amine to form amino radical (NH_2_) groups. Additionally, poly (sodium 4-sterenesulfonate) can be used to enclose carbon nanotubes to graft sulphur trioxide (SO_3_) groups [57]. This occurs via reactions with cysteamine, on the nanocarbon surface, with carboxylic groups to form imidogen (NH) and —sodium hypochlorite (SH) functional groups [58]. Another method that can be applied to increase the MB adsorption capability involves coating charcoal with sodium dodecyl sulfate (SO_3_) groups [98]. For charcoal and chitosan groups, the improvement of MB’s adsorption can be obtained through enhanced numbers of —C=O, —OH, and —NH_2_ [99]. 

## 6. Characterization and Formation of Carbon-Derived Adsorbents

**Figure 5:**
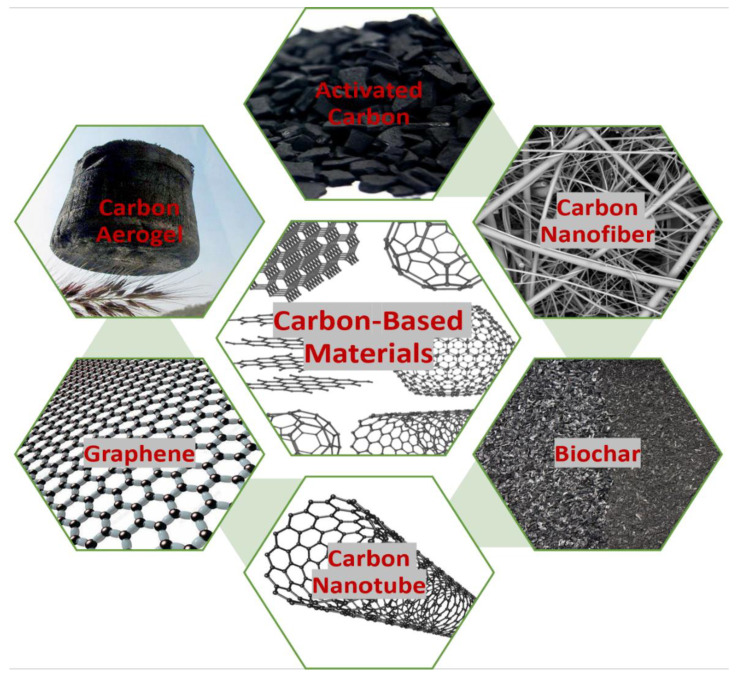
Adsorption procedures used for various carbon-based materials (CBMs).

Adsorption processes are influenced by adsorbent structures, fluid characteristics, the nature of contaminant structures, operating circumstances, and system design features. The adsorbents used for removing impurities from wastewater include biochar, activated carbon, clays, silica gel, composites, zeolites, agro-wastes, and biological and polymeric materials [100]. Most of the pollutants are easily absorbed by carbon-based materials, including hazardous metal ions, medicines, insecticides, metalloids, and other inorganic and organic compounds [101]. The role of adsorbents in water or wastewater treatment is to concentrate and transfer contaminants, thus improving the performance of the process. The reaction also depends on adsorbate–adsorbent interactions. pH, ionic strength, and temperature are the factors that influence the adsorption capability of carbon-based adsorbents [102]. The forces involved in the removal process are hydrogen, van der Waals bonds, covalent and electrostatic interactions, and the hydrophobic effect. Meanwhile, donor–acceptor forces are responsible for the binding and accumulation of chemical compounds on the surfaces of adsorbents [102,103]. These reactions occur in all carbon-based adsorbents including carbon aerogels, carbon nanotubes, carbon nanofibers, and graphene (CAs). The carbon-based materials (CBMs) utilized in adsorption are shown in Figure 5. The advantages and disadvantages of CBMs are tabulated in Table 3.

### Activated Carbons

Recently, activated carbon has been reported to be useful in the remediation of heavy effluents and dye. Activated carbons are generated from commercially available wood, animal-based sources, or coal, and are all natural materials. However, practically any carbonaceous substance can be employed as a precursor in the synthesis of carbon adsorbents [114]. Coal is a widely utilized precursor for activated carbon generation due to its accessibility and low cost [115,116]. Various carbon and mineral combinations emerge from the decomposition of plants to form coal. The sorption qualities and the characteristics of coal are established as a result of the nature, source, and scope of the physical and chemical changes that happen upon deposition. Karaka et al. [117] investigated coal’s use as a dye sorbent. Furthermore, the irregular surface of coal can influence its sorption properties. Peanut shell, [118], bael shell carbon [119], powdered pine cones (both raw and acid-treated) [120], Calotropis procera [121], neem leaf [122], coconut shell [123], and polyvinyl acetate (PVA) alginate super paramagnetic microspheres [124] were successful in reducing the contaminant concentrations of wastewater. Their sorption capacities increased as their adsorbent dosages increased [125].

## 7. Low-Cost Adsorbents

Many variables influence the characteristics of low-cost adsorbents. The precursor should be easily accessible, cheap, and non-toxic. Recent research has focused on natural solids that can remove contaminants from polluted water at cheap cost. Cost is a crucial factor when comparing sorbents. Generally, a sorbent is considered “low cost” if it needs minimal processing, is plentiful in nature, or is a by-product of another business. Many low-cost adsorbents have been employed to remove dyes including agricultural waste, natural materials, and bio-sorbents. Their efficacy in dye removal has been thoroughly investigated. Trash-derived adsorbents have been identified as the most challenging field since they can treat wastewater and reduce waste.

### 7.1. Natural Adsorbent

#### 7.1.1. Clay

Clay is a layered natural adsorbent; with layers including vermiculite, smectites (saponite and montmorillonite), pyrophyllite (talc), mica (illite), kaolinite, serpentine, and sepiolite, clay minerals are accessible [126]. Adsorption occurs as a result of the minerals’ net-negative charge, and this negative charge allows the clay substance to absorb positively charged ions. Their high surface area and porosity account for the majority of their sorption properties [127].

#### 7.1.2. Siliceous

Siliceous is one of the most common materials and reasonably priced adsorbents. It contains glasses, silica beads, alunite, dolomite, and perlite. These minerals were utilized on the basis of the hydrophilic surface’s chemical reactiveness and stability, which was due to a silanol group’s presence. However, special consideration was given to the use of silica beads as adsorbents due to their low resistance to the application of alkaline solutions, limiting their use to media with pH values of less than 8 [73,128,129,130].

#### 7.1.3. Zeolites

Zeolites are aluminosilicate porous materials that naturally form porous aluminosilicates with a variety of cavity configurations linked together by common oxygen atoms. There are numerous species of zeolite [131]. The natural species include chabazite and clinoptilolite. Conversely, clinoptilolite, a heulandite mineral, is the most common investigated substance due to its strong selectivity for specific pollutants. Zeolite has a special characteristic, namely a cage-like structure that is perfect for the elimination of trace pollutants including phenols and heavy metal ions. [132,133].

### 7.2. Bio Adsorbents

Different technologies can be used for the treatment of wastewater that contains dyes. Biological adsorbents that use nonliving biomass have been identified as the most promising approach due to their environmentally safe treatment capability [134]. The effective removal of dyes from the effluent depends on the unique surface chemistry with the presence of different functional groups in the cell wall of microorganisms, such as alcohol, aldehydes, ketones, carboxylic, ether, and phenolic compounds, which make the bio-sorbents have a high affinity toward dye and are attractive materials for dye removal [135]. Biological materials including chitin, peat, chitosan, yeast, and fungi biomass are frequently used in the sorption of dye from the solution through the mechanism of chelation and complexion [136]. A good adsorbent used in the removal of dye must have several desirable properties, including a large surface area, high adsorption capacity, large porosity, easy availability, stability, feasibility, compatibility, eco-friendliness, and ease of regeneration, as well as being highly selective in terms of removing different varieties of dyes [137]. The pore volume of the bio adsorbents and the functional groups of dyes are the deciding factors in the achievement of high dye adsorption. The presence of a large pore volume allows the binding of the highest number of dye molecules to the adsorbent [92]. Higher surface area, higher porosity, and low ash content lead to high adsorption capacity. Functional groups (hydroxyl, carboxyl, etc.) on the surface of biomass-based adsorbents are important properties determining the hydrophobicity or hydrophilicity of biochar as well as their adsorptive mechanism [138]. Likewise, the diversity of microbes consisting of different species of bacteria, fungi, yeast, and algae was studied in relation to the removal of dye molecules [139]. Besides the high sorption capacity toward dye, the dye removal performance can be improved by combining the biosorption process with the biodegradation processes using living cells [140]. The pH, bio-sorbent dose, initial dye concentration, temperature, and contact time are the influencing factors for the biosorption capacities of biomass [141].

#### 7.2.1. Bacterial

Bacteria can play a role in bioremediation processes by adsorbing pollutants from aqueous media through a variety of methods, including dead biomasses [142]. Due to their tiny size, widespread distribution, and capacity to grow in a variety of environmental circumstances, they make excellent adsorbents [143]. Bacterial species were identified to successfully adsorb reactive dyes from wastewater under optimal environmental conditions [144]. The rates of bacterial dye decolorization vary according to the bacterium type, dye reactivity, and operational factors such as temperature, pH, co-substrate, electron donor, and dissolved oxygen content. It is possible to successfully treat textile dyes using extremophiles. According to the Langmuir adsorption isotherm, the maximum solubility capacity of basic blue dye is 139.74 mg/g. Carboxyl and phosphonate groups that are present on adsorbent surfaces may operate as possible surface functional groups, which are capable of binding cationic contaminants [145]. Numerous functional groups on the surface of the Penaeus indicus biomass were probably involved in the binding of the Acid Blue 25 dye, although the amino groups and alpha-chitin were by far the most significant [146]. Bacillus subtilis was immobilized on a calcium alginate bead and then used in batch and continuous reactors to remove MB. The kinetic analysis of the batch and continuous contactors revealed a removal rate of more than 90% [147]. Additionally, bacteria were adapted for MB’s removal using electro-spun nanofibrous-encapsulated cells (Sarioglu et al., 2017a). Due to their variable cell wall compositions, biosorption fidelity is dependent not only on the group of ions but also on the type of bacteria.

#### 7.2.2. Fungal

Fungal biomasses include sugars, proteins, and lipids, as well as functional groups (alcohols, carboxyls, and alkanes), which provide them with specific qualities and uses in wastewater treatment [148]. The biotreatment of dye-containing wastewater effluent by fungal cells was reported to be cost-effective, simple to implement, environmentally benign, and devoid of nutritional requirements [149]. Numerous fungi have been applied as effective candidates for the removal of a variety of dyes from effluents, including *Trichoderma* sp. [149], *Sarocladium* sp. [150], growing *Rhizopus arrhizus* [151], and several varieties of white-rot fungi [152]. It was shown that the removal rate of anionic dyes increases whereas the removal rate of cationic dyes decreases in low-pH solutions. In contrast, a high-pH solution enhances the removal of cationic dyes and results in a low proportion of anionic colors being removed [148]. The point of zero charges (pHpzc) is a critical metric for understanding the adsorption mechanism and its favorability. The pHpzc value provides information on the active sites and adsorption capacity of adsorbents. When the pH is larger than the pHpzc, cationic dye adsorption is more advantageous owing to the presence of functional groups (OH^−^, COO^−^), but anionic dye adsorption is more favorable when the pH is less than the pHpzc due to the positively charged surfaces of the adsorbents [95]. In general, the use of fungal biomass as a dye decolorizer and adsorbent is a viable alternative to existing technology. Along with the regulation of environmental factors, it is critical to consider the genotype and preparation of the biomass in order to ensure optimum dye-adsorption performance.

#### 7.2.3. Algae

Algae are one of the best sources of bio-sorbents since they have high biosorption ability and are readily accessible [153]. The algal cell wall is composed of polysaccharides, including xylan, mannan, alginic acid, and chitin. In addition to proteins, these components may include amino, amine, hydroxyl and imidazole, phosphate, and sulfate groups [143]. Pretreatments such as encapsulation and surface modification may improve the sorption capacity of algae. The adsorption ability of citric acid-functionalized brown algae for textile dye (crystal violet) removal in aqueous solutions was investigated. It was found to improve the uptake capacity by up to 279.14 mg/g [154]. This process was also due to electrostatic interactions.

The adsorption of five water-soluble dyes was performed using magnetically sensitive brown algae (Sargassum horneri). Using microwave-synthesized iron oxide nano- and microparticles, the magnetic modification allowed for quick and selective separation [155]. After 2 h contact time, the sorbent demonstrated maximal acridine orange sorption capacity (193.8 mg/g) but not malachite green sorption capacity (110.4 mg/g). Sargassum macroalgae are frequently investigated for their ability to remove colors. MB is a popular dye that is removed by dye species. Anionic dyes are eliminated in acid, and cationic dyes (e.g., MB) are eliminated in alkaline. This is because hydrogen (H+) ions are involved in the biomass–pollutant interaction mechanism. To reduce the quantity of absorbed dye, the adsorbent’s surface might be charged positively to compete with the dye’s cations. At increased pH, carboxyl groups have a negative charge, resulting in the electrostatic binding of cationic dyes. Other criteria that influence the biosorption efficiency include the processing of the biomass into the adsorbent, the starting contaminant concentration, and the biomass dose, temperature, and contact duration.

#### 7.2.4. Yeast

Yeast is a single-celled organism that has numerous advantages over filamentous fungus in terms of the adsorption and accumulation of pollutants, as well as its growth rate, decolorization rate, and ability to live in harsh settings [156]. The carboxyl hydroxide, polymer, amino, and phosphate functional groups on the yeast surface alter the pH of the tested solution [157]. Yeast biomass has been shown to bio-adsorb several types of colors. The bio-sorption process is affected by the pH, pollutant concentration, yeast mass, temperature, and contact duration [158]. Reactive Blue 19 (RB 19) and Red 141 (RR 141) were studied in Antarctic yeast (Debaryomyces hansenii F39A). At pH 6.0, with 100 mg/L as the initial dye concentration, and a 2 g/L biomass dose, 90% of RR 141 and 50% of RB 19 were adsorbed. However, at a 6 g/L biomass dose, 90% of RB 19 was adsorbed. The Langmuir isotherm was defined as the pseudo-second-order kinetics for each dye system, and the Langmuir isotherm was the best-matched model [159].

The removal of Reactive Blue 160 dye using residual yeast and diatomaceous earth (RB 160) was also investigated. The dye removal capability of the two bio-sorbents was 8.66 mg/g and 7.96 mg/g at pH 2 [160]. The biomass functional group’s positive charge interacted with the anionic dye. The yeast biosorption data were better fitted to the Freundlich isotherm model, whereas the diatomaceous earth data were better fitted to the Langmuir isotherm. Another study used brewer’s yeast biomass that was able to adsorb the basic dyes safranin O (SO), MB, and malachite green (MG)) from aqueous solutions within 1 h. This study also reported that MB’s and MG’s adsorption kinetics were pseudo-second-order, whereas those of SO were pseudo-first-order. Yeast was also found to adsorb hydroxyl, cyano, and other functional groups [161].

### 7.3. Agricultural and Industrial Materials’ Adsorbents

#### 7.3.1. Agricultural Waste and Plant Adsorbents

The use of agricultural wastes and plants to adsorb organic and inorganic contaminants is considered to be a viable alternative to standard wastewater treatment procedures [162]. Numerous investigations on the elimination of MB have recently been conducted, which involved employing dead or living agro-waste, algae, fungi, and a variety of naturally occurring and low-cost agro-waste sources as adsorbents, including fruit peels, seeds, leaves, straw, sawdust, bark, sludge, and ash [163].

Numerous studies demonstrated that the dye-adsorption properties of certain biomasses are highly dependent on the kind of dyes used, and the processing procedures used were successful in reducing the contaminant concentrations of wastewater. This group of biological compounds of agro-waste-derived adsorbents was capable of collecting and concentrating dyes in aqueous solutions. Due to the non-selective nature of these biomaterials, all pollutants, both target and non-target, became concentrated on the adsorbent’s surface, providing significant removal for the purpose of pollution control. The technique allows the adsorption of only those ions for which it has a particular affinity. In comparison to other methods, bioadsorption is rated as preferable due to its low cost, simplified design, great efficiency, and capacity to separate a wide variety of contaminants [164].

#### 7.3.2. Industrial Products

Fly ash, metal hydroxide sludge, bio solids, red mud, and waste slurry are examples of industrial products that may be employed as dye adsorbents since they are low-cost and readily available. Adsorbents made from industrial waste may be used instead of more expensive traditional adsorbents [165].

##### Fly Ash

Fly ash is a type of industrial waste that may be used to adsorb dyes. Fly ash is generated in enormous quantities during combustion operations and may include certain harmful chemicals, such as heavy metals [166]. However, bagasse fly ash, created in the sugar industry, is devoid of hazardous metals and is often employed for color adsorption. Its qualities are very variable and are dependent on its source. Adsorption investigations were conducted on congo red and MB textile dye solutions and it was discovered that the monolayer development on the adsorbent surface and the adsorption process are exothermic in nature. Fly ash from thermal power plants may be efficiently utilized as an adsorbent to remove colors from dyeing industry effluents [167]. The removal of methylene blue, using fly ash as an absorbent, was investigated and a maximum removal of 58.24 percent was reported at pH 6.75 and 900 mg/l adsorbent for an initial methylene blue dye concentration of 65 mg/l. At various beginning conditions, fly ash could remove 95–99 percent of the dye from the solution, and the Langmuir constant *q_m_* was 1.91 mg g–1 and the *K_a_* value was 48.94 L mg–1 with a liner regression coefficient of 0.999 [168].

##### Metal Hydroxide Sludge

Sludge made from metal hydroxide is used to clean up azo dyes. It has insoluble metal hydroxides and salts. Researchers discovered that at 30 °C and pH 8–9, electroplating industrial hydroxide sludge had maximal adsorption capacities of 45.87 and 61.73 mg/g for Reactive Red 120 and Reactive Red 2, respectively. The pH also influenced the adsorption and development of dye–metal complexes. Sludge of metal hydroxide was used as an adsorbent and it was found to have a maximum adsorption capacity of 270.8 mg/g at 30 °C and an initial pH of 10.4. Metal hydroxide, as a low-cost adsorbent for the removal of the Remazol Brilliant Blue reactive dye from a solution, was reported to have a 91.0 mg/g monolayer adsorption capacity at 25 °C and pH 7 [169].

##### Red Mud

Red mud is another industrial byproduct and bauxite manufacturing waste product used to make alumina. The capacity of discarded red mud as an adsorbent for the removal of dye from its solution was examined, and it was found to be effective. It was found that the greatest dye removal via adsorption occurred at pH 2, and this was followed by the Freundlich isotherm [170]. Red mud was used as an adsorbent to remove a basic dye, methylene blue, from its aqueous solution. The adsorption capacity of red mud was determined to be 7.8 × 10^−6^ mol/g. The use of discarded red mud as an adsorbent was shown in order to extract congo red from aqueous solution. The dye-adsorption capability of the red mud was determined to be 4.05 mg/g. Using acid-activated red mud, the adsorption of congo red from wastewater was examined [171]. The Langmuir isotherm provided the greatest match to the experimental data. Using red mud, the removal of methylene blue, quick green, and rhodamine B from wastewater was investigated. Fast green, Methylene blue, and rhodamine B were removed with red mud at percentages of 75.0, 94.0, and 92.5, respectively; the adsorption process followed both the Langmuir and Freundlich isotherms and was exothermic in nature [172].

### 7.4. Activated Carbon-Based Adsorbent Derived from Low-Cost Waste

**Figure 6:**
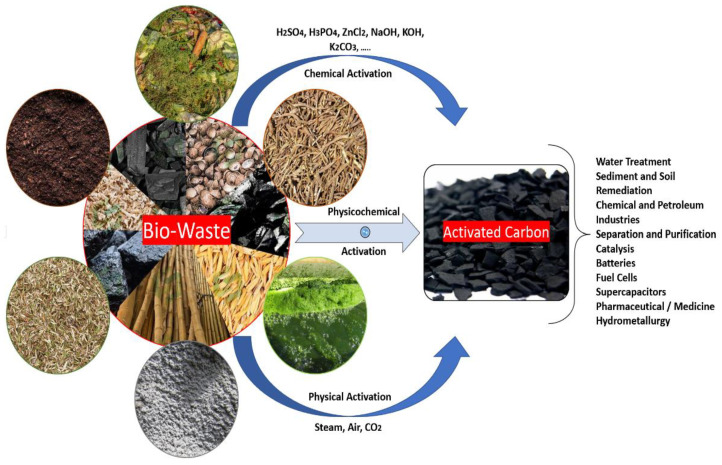
Schematic clarification of activated carbon derived from bio-waste and its potential uses.

Agricultural wastes are rich in hemicellulose, cellulose, and lignin. Their surfaces are covered with a variety of active groups, including carboxyl, hydroxyl, methyl, and amino [95]. These functional groups may adsorb dyes in a variety of ways, including via complexation, hydrogen bonding, and ion exchange [152]. Table 4, Table 5, Table 6, Table 7, Table 8, Table 9, Table 10, Table 11, Table 12 and Table 13 highlight different agricultural and forest waste types, their biosorption capacity, and the activation reagents required. Numerous acids have been utilized to activate biosorbents to increase their binding sites, aqueous solution chemistry, specific surface area, and porosity. Phosphoric acid increases the bond-breaking process in agricultural waste biomass, thereby boosting its carbon output [173]. Sodium hydroxide (NaOH), sulphuric acid (H_2_SO_4_), and potassium hydroide (KOH) are often utilized as activators in the manufacturing of agricultural waste-based bioadsorbents (Table 4, Table 5, Table 6, Table 7, Table 8, Table 9, Table 10, Table 11, Table 12 and Table 13). Numerous environmental factors, including the adsorbent dosage, temperature, contact time, solution pH, particle size of the plant-based adsorbent, agitation, and initial dye concentration, all have a significant influence on the biosorption process. The pH of the solution, the particle size of the plant-based adsorbent, the rate of agitation, and the initial dye concentration all have a substantial effect on the biosorption process. The pH of a solution has an effect on both the aqueous solution’s chemistry and the binding sites on the surfaces of the adsorbents [174]. Due to the abundance of low-cost products, they constitute excellent raw materials for the manufacturing of activated carbon. Table 4, Table 5, Table 6, Table 7, Table 8, Table 9, Table 10, Table 11, Table 12 and Table 13 summarize the different types of activated carbon derived from biomass and their maximal adsorption capacities for MB elimination. A schematic clarification of bio-waste-derived adsorbents is shown in Figure 6.

#### Isotherm Equilibrium and Sorption Capacity of Biowaste-Derived Adsorbents

Table 4, Table 5, Table 6, Table 7, Table 8, Table 9, Table 10, Table 11, Table 12 and Table 13 show the outstanding capabilities and operating conditions of bio-waste-derived adsorbents with high sorption capacities that have been established over the last decade. Furthermore, this review sought to enclose a broad range of recent research on unconventional adsorbents to educate researchers about the design parameters and sorption capacities for the adsorption of various bio-waste materials. Phosphoric acid improved dye biosorption by grafting phosphate functions onto the biomass and enhancing the acid functions involved in dye fixation

Previous studies addressed equilibrium isotherms and kinetic features by employing models ranging from Henry’s law to the Langmuir (monolayer), Redlich–Peterson, Sips, and Freundlich models for fitness analyses. The kinetic and isotherm models are useful predictive tools for adsorbent system regeneration, design parameter optimization, and adsorption and desorption capacity maximization, and can, by these means, optimize waste disposal. Additionally, most of the previous studies were conducted in batch mode, which enables more cost-efficient and effective treatments for the design of continuous systems. The adsorption capacity of an adsorbent can be determined using equilibrium isotherms. Equilibrium isotherms link the equilibrium concentration of the adsorbate (*Ce*) to the quantity of the adsorbent (*qe*). Furthermore, the adsorbate characteristics and adsorbent surfaces can be studied in detail using liquid–solid isotherms.

Table 4, Table 5, Table 6, Table 7, Table 8, Table 9, Table 10, Table 11, Table 12 and Table 13 illustrate the operating conditions, sorption capacities, and appropriate kinetic isotherms for adsorbents derived from bio-waste over the last decade. Furthermore, this review sought to enclose a broad range of recent research on unconventional adsorbents in order to educate researchers about the design parameters and sorption capacities related to the adsorption of various bio-waste materials [175,176,177].

**Table 4:** Summary of bio-waste-derived adsorbent studies in 2012.

Biosorbents	Q_max_ (mg/g)	Most Appropriate Model	pH	Temperature (°C)	Time (min)	Reference
Pink Guava leaf	250	L-K2	NA	30	300	[178]
Malted sorghum mash	357.1	L	7.3	33	18	[179]
Rice husk	8.13	L-K2	5.2	25	NA	[180]
Water Hyacinth	8.04	L-K2	8	25	80	[181]
Date stones	398.19	S-K2	7	30	270	[182]
Oil palm shell	133.13	NA	NA	30	10	[183]
Swede rape straw	246.4	L	NA	25	NA	[184]
Pyrolysis of wheat	12.03	S	8–9	20	50	[185]

**Table 5:** Summary of bio-waste-derived adsorbent studies in 2013.

Biosorbents	Q_max_ (mg/g)	Most Appropriate Model	pH	Temperature (°C)	Time (min)	Reference
Pea shells	246.91	L	2–11.5	25	180	[185]
Coconut fiber	500	L-K2	7.8	30	30	[186]
Papaya leaves	231.65	L	2–10	30	300	[187]
Untreated Alfa grass	200	L-K2	12	20	180	[188]
Neem leaf Powder	401.6, 352.6	F-K2	7	87	60	[189]
Corn husk	662.25	F	4	25	120	[190]
Lagerstroemia microcarpa	229.8	L-K2	NA	30	360	[191]
watermelon (Citrullus lanatus)	489.80	L-K2	NA	30	30	[192]
Sugarcane bagasse	95.19%	NA	8.76	25	193	[193]

**Table 6:** Summary of bio-waste-derived adsorbent studies in 2014.

Biosorbents	Q_max_ (mg/g)	Most Appropriate Model	pH	Temperature (°C)	Time (min)	Reference
Iron oxide-modified montmorillonite	69.11	L-K2	8	35	240	[194]
Magnetic NaY Zeolite	2.046	L	10.3	50	45	[195]
Fe_3_O_4_ graphene/MWCNTs	65.79	L-K2	7	10	30	[196]
Water hyacinth	111.1	L	8-10	30	300	[197]
Lantana camara stem	19.84	F-K2	3-11	20	60	[198]
Natural peach gum (PG)	298	L-K2	6-10	25	30	[199]
Activated fly ash (AFSH)	14.28	F-K2	3.0-10.0	20	100	[200]

**Table 7:** Summary of bio-waste-derived adsorbent studies in 2015.

Biosorbents	Q_max_ (mg/g)	Most Appropriate Model	pH	Temperature (°C)	Time (min)	Reference
Magnetic biochar derived from empty fruit bunch	31.25	L-K2	2-10	25	120	[201]
Magnetic adsorbent derived from corncob	163.93	L-K2	NA	25	500	[202]
Fe_3_O_4_ bentonite	NA	K2	7	NA	20	[203]
Magnetic chitosan/organic rectorite	24.69	L-K2	6	25	60	[204]
Poly acrylic acid/MnFe_2_O_4_	NA	K2	8.3	25	NA	[205]
Fe_3_O_4_ xylan/poly acrylic acid	438.6	L-K2	8	25	NA	[206]
Fe_3_O_4_ modified graphene sponge	526	L-K2	6	NA	NA	[207]
Xanthate/Fe_3_O_4_ graphene oxide	714.3	L-K2	5.5	25	120	[208]
Magnetic carbonate hydroxyapatite/ graphene oxide	405.4	L-K2	9.1	25	90	[209]

**Table 8:** Summary of bio-waste-derived adsorbent studies in 2016.

Biosorbents	Q_max_ (mg/g)	Most Appropriate Model	pH	Temperature (°C)	Time (min)	Reference
Palm shell	163.3	F-K2	NA	25	NA	[210]
Fe_3_O_4_-activated montmorillonite	106.38	L-K2	7.37	20	25	[211]
Clay (montmorillonite and vermaculti)/polyaniline/Fe_3_O_4_	184.5	L-K2	6.3	25	30	[212]
Magnetic chitosan/active charcoal	200	L-K2	7.73	25	200	[99]
Fe_3_O_4_ /poly acrylic acid	73.8	L-K2	NA	45	NA	[213]
Magnetized graphene oxide	306.5	L-K2	9	25	360	[214]
Corn straw	267.38	F-K2	8	25	20	[215]
Magnetic chitosan and graphene oxide	243.31	K2-L	12	60	60	[216]

**Table 9:** Summary of bio-waste-derived adsorbent studies in 2017.

Biosorbents	Q_max_ (mg/g)	Most Appropriate Model	pH	Temperature (°C)	Time(min)	Reference
Corn shell	357.1	L	4	25	30	[217]
Magnetic activated carbon	2.046	F-K2	10	25	120	[218]
Magnetic halloysite nanotube nano-hybrid	689.66	L-K2	10	25	180	[219]
Magnetic polyvinyl alcohol/laponite RD	251	L-K2	5.5	25	60	[220]
Aegle marmelos leaves	500	F-K2	6	25	120	[221]
Oak-acorn peel	109.43	L-K2	7	24	120	[222]
Geopolymers	15.95-20.22	S-K2	4-12	25	80	[223]
Ouricuri fiber	31.7	S-K2	5.5	25	5	[224]

**Table 10:** Summary of bio-waste-derived adsorbent studies in 2018.

Biosorbents	Q_max_ (mg/g)	Most Appropriate Model	pH	Temperature (°C)	Time (min)	Reference
Carboxymethyl/cellulose/ Fe_3_O_4_/SiO_2_	31.02	L-K1	11	NA	60	[225]
Cellulose-grafted	7.5	L	8		5.5	[226]
NiFe_2_O_4_Ca/alginate	1243	R-K1	6.5	25	180	[227]
Magnetic alginate	161	L	7	20	120	[228]
Magnetic hydrogel Nanocomposite of poly acrylic acid	507.7	L-K1	7	25	120	[229]
Magnetized graphene oxide	232.56	L-K2	9	30	10	[230]
Soursop	55.397	R-K2	5.5	25	300	[231]
Sugarcane Bagasse	17.434	S-K2	5.5	25	300	[231]
Palm sawdust	53.476	F-K2	8	25	120	[232]
Eucalyptus sawdust	99.009	F-K2	6	20	60	[232]

**Table 11:** Summary of bio-waste-derived adsorbent studies in 2019.

Biosorbents	Q_max_ (mg/g)	Most Appropriate Model	pH	Temperature (°C)	Time (min)	Reference
Fir bark	330.00	F-K2	NA	25	40	[233]
Pumpkin peel	198.15	L-K2	7	50	180	[234]
Rice husk	608	L	7	25	60	[235]
date stones	163.67	F-K2	10	25	360	[236]
Seaweed	1279.00	L-K2	4	25	50	[237]
Moroccan cactus	14.04	L	5	25	60	[238]
Syagrus oleracea	893.78	L-K2	7	25	20	[239]
Mentha plant	588.24	L	10	25	30	[240]
Palm leaf	500	L	2	30-60	30	[241]

**Table 12:** Summary of bio-waste-derived adsorbent studies in 2020.

Biosorbents	Qmax (mg/g)	Most Appropriate Model	pH	Temperature (°C)	Time (min)	Reference
Kendu fruit peel	144.90	L-K2	6	25	100	[242]
Magnesium oxide nanoparticles	163.87	L-K2	7.3	25	70	[243]
Fava bean peel	140.00	L	5.8	27	NA	[244]
Dicarboxymethyl cellulose	887.60	L-K2	3	25	60	[245]
Alginate-based beads	400.00	L-K1	7	25	NA	[246]
Black cumin seeds	16.85	F-K2	4.8	25	20	[247]
Dragon fruit peels	195.2	L-K1	3-10	50	60	[248]
Litsea glutinosa seeds	29.03	L-K2	9	40	600	[249]
Moringa oleifera leaf	136.99	F-K2	7	25	90	[250]

**Table 13:** Summary of bio-waste-derived adsorbent studies in 2021.

Biosorbents	Q_max_ (mg/g)	Most Appropriate Model	pH	Temperature (°C)	Time (min)	Reference
Grass waste	364.2	L	10	45	15	[251]
Mangosteen peel	871.49	L-K2	10	25	60	[252]
Coconut shell	156.25	F-K2	4.9	25	360	[253]
Core shell	34.3	L-K2	7	25	120	[254]
Banana stem	101.01	F-K2	7	25	90	[255]
Alginate beads	769	L-K2	8	30	NA	[256]
Ulva lactuca	344.83	L-K2	11	25	NA	[257]
Cassava Stem	384.61	L-K2	9.2	25	60	[258]
Corncob	864.58	L-K2	5	25	360	[259]

**General equation**

(1) $$Q_{\max}=\frac{\left(C_{0}-C_{e}\right)V}{W}$$

where *V* is the solution volume (L) and *W* is the adsorbent mass (mg/L) (g), *C*_0_ and *C_e_* are the initial and equilibrium dye concentration in mg/L, respectively.

**Langmuir (L) isotherm model:**

(2) $$qe=\frac{Q{}^{\circ}\cdot K\cdot C_{e}}{1+K\cdot C_{e}}$$

where *qe* is the adsorbate quantity per unit of adsorbent (mg/mg), *C_e_* is the equilibrium concentration of the adsorbate (mg), *K* is the Langmuir adsorption coefficient (mg/g) (L/mg) ^1/*n*^.

**Freundlich (F) isotherm model:**

(3) $$qe=\mathrm{Kf}\;{C_{e}}^{1/n}$$

where *qe* is the quantity of adsorbates per unit of adsorbent (mg/g), *C_e_* is the adsorbate equilibrium concentration in the solution (mg), *n* is the empirical coefficient, Kf is the Freundlich adsorption coefficient (mg/g) (L/mg) ^1/*n*^.

**Redlich–Peterson (R) isotherm model:**

(4) $$qe=\frac{K_{R}C_{e}}{1+a_{R\;}C_{e}^{g}}$$

where *qe* is the adsorbate quantity per unit of adsorbent (mg/mg), *K_R_* (L g^−1^) and *a_R_* (L*^g^*·mg*^−g^*) are constants, *C_e_* is the equilibrium concentration of the adsorbate (mg), *g* is the exponent (0 ≤ g ≤ 1).

**Sips (S) isotherm model:**

(5) $$qe=\frac{q_{ms}K_{s}C_{e}^{n_{s}}}{1+K_{s}C_{e}^{n_{s}}}$$

where *q_ms_* is the maximum adsorbed amount (mg/g), *K*_s_ (L*^ns^*·mg^−*ns*^) and *n*_s_ are the Sips constants, *C_e_* is the equilibrium concentration of adsorbate (mg), *qe* is the quantity of adsorbate per unit of adsorbent (mg/mg).

**Modeling adsorption kinetics:**

Adsorption kinetics were used to explore the pace and mechanisms of adsorption, which may occur due to physical and chemical events, and to compare these with experimental data.

**Pseudo-First-Order Kinetics (K1):**

ln (*Q_e_* − *Q_t_*) = ln *Q_t_* − *k*_1_*t* (6)

where *Q_t_* is the adsorbed amount at time *t*, *Q_e_* is the equilibrium amount, *t* is the time in minutes, and *k*_1_ is the rate constant.

**Pseudo-Second-Order Kinetics (K2):**

(7) $$\frac{t}{Qt}=\frac{1}{k_{2}Q_{e}^{2}}+\left(\frac{1}{Q_{e}}\right)t$$

where *Q_t_* is the adsorbed amount at time *t*, *Q_e_* is the equilibrium amount, *t* is the time in minutes, and *k*_2_ is the rate constant.

## 8. Cost Analysis of Adsorbents

Several authors indicated that the application of bio adsorbents derived from microorganisms and forest and agricultural waste is lower than the cost of traditional treatment methods. Nonetheless, none of these research works considered the cost analysis in their final assessment. For a cost effective system, the volume of adsorbent used, the simplicity of preparation or processing, green chemistry ideas, and the activation process used are the factors that need to be considered [260]. In contrast, another study emphasized the term “low-cost adsorbents” to refer to their initial costs, and their local availability, transportation, treatment process, recycling, and lifespan concerns, as well as regenerating and treatment methods [95]. Additionally, most of the previous research works on biomass-based adsorption were undertaken on a laboratory scale using simulated wastewater, thereby restricting the cost of the analysis to be undertaken.

The ability to remove Basic Red 09 dye from wastewater was investigated using coconut shell, groundnut shell and rice husk. The study revealed the cost of 1 g of adsorbent used to remove 4.54, 0.91, and 0.97g when operational expenses such as production, maintenance, feedstock, transportation, labor, and distribution costs are included [261]. Groundnut shell-based biochar showed the highest adsorption capacity (46.3 mg/g) and the lowest cost-per-unit in grams of Basic Red 09 dye removal (0.91). A phosphoric acid-functionalized locust bean pod adsorbent was produced for the removal of RhB dye, and the initial cost of this adsorbent was determined. They revealed that the activated carbon generated by these plant sources was roughly six times less costly than conventional activated carbon. The expense is mostly borne by phosphoric acid and deionized water [262].

## 9. Regeneration and Economic Challenges of Bio-Waste-Derived Adsorbents

The desorption process can induce the application of reused adsorbent, thus reducing waste and minimizing capital and operational costs [95]. Common desorption methods include thermal, acid (i.e., hydrochloric acid (HCl), H_2_SO_4_, phosphoric acid (H_3_PO_4_), and nitric acid (HNO_3_)) NaOH, organic solvent (methanol), vacuum, and biological methods [92]. Solvent desorption through drying processes can vaporize and remove the dye with suitable a combination ratio between the adsorbent and the solvent. [95]. On top of reuse or regeneration of the adsorbent, the selection of an appropriate adsorbent, particularly at large scales, plays a vital role in terms of ensuring an efficient and economical treatment method. Powdered activated carbons were reported as being inappropriate for industrial applications due to their high costs and times, and complex recovery processes [263]. This can lead to high energy consumption due to inefficient processes. However, the post-treatment of adsorbent and effluent that contains contaminants is necessary after dye’s removal from wastewater [264]. Immobilization and stabilization immobilization are two possible ways of securely disposing of the final effluent; for example, utilizing in concrete technology as a binder material [265,266].

Dahiru et al. [267] reported that the efficiency of banana peel adsorbent reduced to 64% after 5 uses. Despite there being numerous studies on the development of bio-waste-derived activated carbon, there were minimal efforts focused on the technoeconomic assessment and life cycle analysis of these applications. This signifies the need for the development of adsorbents that are more robust in order to maintain removal rates, optimize costs, and promote the sustainable regeneration of adsorbents.

## 10. Management of Post-Adsorption Materials

**Figure 7:**
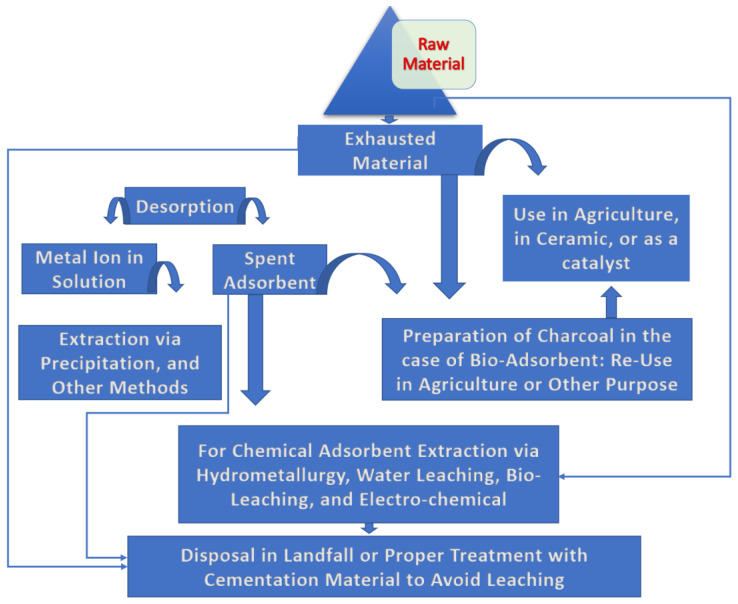
Adsorbent disposal management after adsorption.

After usage, the adsorbent can be managed in a variety of ways, including regeneration, re-use, and safe disposal (Figure 7). Regeneration may be accomplished in a variety of ways, including with a chelating desorbing agent, an alkali desorbing agent, a salt desorbing agent, or via thermal regeneration [268]. In addition to the forementioned approaches, organic pollutants may be regenerated via ultrasonic regeneration, microbiological regeneration, microwave-assisted regeneration, thermal regeneration, chemical regeneration, ozonation, photo-assisted oxidation, and electrochemical oxidation [269]. After many adsorption–regeneration cycles, the adsorbent’s efficacy decreases [270]. After many adsorption–regeneration cycles with the same pollutant, the method renders the adsorbent redundant. The used adsorbent may be disposed of in a landfill or burnt or recycled [271]. Prior to landfill disposal, used adsorbents containing hazardous elements can be stabilized/solidified [272], thus increasing the expense of the adsorbent’s life cycle evaluation. Enhancing the adsorbent’s sustainability may be accomplished by properly disposing and reusing it in other applications. The used adsorbent can be used in a variety of ways, including as a catalyst [273], in brick formulations [274], in road construction [275], or in cement clinkers [276]. The three major applications of wasted adsorbents are as follows: as a catalyzer, in the manufacturing of ceramics, and as a fertilizer.

### 10.1. Application as a Catalyst

Following the adsorption process, the used adsorbents can be employed as catalysts throughout the processes of photodegradation [277], nitrophenol’s reduction to aminophenol [278], hydrocarbon oxidation [277], the conversion of xylose and xylan to furfural, and also the conversion of phenylacetylene to acetophenone [279]. Depending on the type of pollutant, the final product can be further analyzed using nuclear magnetic resonance (NMR) spectroscopy, high-performance liquid chromatography (HPLC) [279], gas chromatography [273], ultra-violet spectroscopy [278], and Fourier-transform infrared spectroscopy (FTIR) [277]. In some cases, the catalytic activity of the metal ion varies according to its position on the adsorbent, the conversion and selectivity inside the oxidation of cyclohexanol, as well as the increasing of ethyl benzene [280]. Despite the vast potential of expended adsorbents to induce catalysis, several issues must be addressed, with the most significant being the leachability of the pollutant or other materials from the adsorbent during their usage as catalysts. The majority of research employed either the California waste removal test or the Toxicity Characteristic Leaching Procedure (TCLP) for leaching measurement [281]. In many situations, wasted adsorbent contains dangerous elements, and environmental organizations (e.g., the USEPA in the United States, the CPCB in India, and DEFRA in the United Kingdom) enforce strict disposal rules. Consequently, this problem can be eliminated by increasing the use of nontoxic waste adsorbents [282].

### 10.2. Application in Ceramic Production

Used adsorbents can also be employed as ingredients in the manufacturing of ceramic materials, including as fillers in the cement industry. The issue of the adsorbent’s hazardous nature may be mitigated by its application in the manufacturing of ceramics as well as in road building. The leaching of dangerous materials from the used adsorbent can be managed with the correct preparation conditions. Spent adsorbent (zeolite- and perlite-supported magnetite following molybdenum adsorption) was combined with sludge at a ratio of 3/97, which corresponded to the adsorption capacity of the loaded adsorbent [283]. Additionally, ceramic products may help in preventing the leaching of additional heavy metals (including Nickel, Chromium, Copper, Zinc, Arsenic and Cadmium) that spike during the application of the ceramic synthesis technique. This is advantageous in the treatment of polluted eluent generated during desorption operations. Additionally, the used adsorbent may be disposed of by immobilizing it inside the phosphoric glass matrix. It was also shown that around 20% of wasted adsorbent can be integrated during glass production [284].

### 10.3. Application as Fertilizer

The used adsorbent can be converted into a user-friendly material, including fertilizers. The properties required for fertilizer production include affinities for anions and cations and long-term stability in various environments. Charcoal is mainly used as a fertilizer. Calcium (Ca), Nitrogen (N), Potassium (K), and Phosphorus (P) are abundant in biomass. By applying this method, nutrients are returned to the soil, potentially improving soil fertility [285]. The use of biodegradable organic adsorbents as fertilizers is possible. It was reported that 20 days is required for carboxy methyl cellulose, a copper-removing chitosan, to break down [286].

The pyrolysis of discarded bio-sorbent, occurring as a result of the adsorption of contaminants in biochar or direct soil applications, yields charcoal, biochar, and a variety of products, each with a distinct economic value [287]. Toxic substances that are present in soil can be reduced by adding charcoal. The use of charcoal (15 g/kg) was found to lower chromium and cadmium contents in a plant by 33.50 and 28.73 percent, respectively [288]. Additionally, crops need nitrates and phosphates for their growth, and charcoal is inefficient in terms of serving these needs. Consequently, metal ions such as Ca, Mg, and Al may be added to charcoal [289]. In the case of phosphate, these components increased the formation of H bonds or precipitation, whereas in the case of nitrate, they increased the electrostatic attraction [289]. Meanwhile, the nonfuel fraction gases (carbon monoxide (CO), methane (CH_4_), and other hydrocarbons) may be utilized to synthesize various chemical reagents in order to synthesize biofuels [290]. Additionally, the use of adsorbents as fertilizer can improve metal sequestration [291], improvement of soil’s nutritive value [292], increased soil organic carbon (SOC) (due to the application of activated carbon) [282], and increased water-holding capacity of the soil [285]. The content of each heavy metal in charcoal has a specific threshold level. Lead concentrations in basic and premium biochar should be lower than 120 and 150 g/t. This includes the need for higher charcoal demands as compared to commercial fertilizer, and the regulated release of nutrients to prevent soil contamination as well as metal ion accumulation. This has influenced the initial capital cost of recovering all products from pyrolysis, such as heat and gases during biomass feedstocks [292].

## 11. Cost-Effectiveness: Desorption versus Disposal

Following adsorption process, adsorbents may be desorbed and renewed until the pollutant content in the effluent is maintained below the permitted level established by regulatory bodies. The used adsorbent may be repurposed for different applications such as catalyst synthesis, ceramic manufacture, and pollutant removal, or it can be discarded. The desorption of pollutants may be accomplished using an alkali or acid reagent, a chelating agent, or salt; or, for organic pollutants, chemical, thermal, microwave, or other processes can be used [293]. Alkali was reported as the most effective method for removing heavy metals from chemical-based adsorbents (Table 14). The employment of an acid, an alkali, a chelating molecule, or a chemical as a desorbing agent result in waste creation (secondary pollution) in contaminated eluent. As a result, this approach suffers from the same disposal issues as other approaches, such as wasted adsorbent, which have environmental and economic implications. Nonetheless, there are rare occasions when metals that are laced with other heavy metals can be recovered, such as chromium (Cr) being recovered from barium chloride (baCl), and mercury (Hg) from the ethylenediaminetetraacetic acid (EDTA)–Hg combination being recovered as mercury chloride (HgCl) [294].

## 12. Limitations and Strategies

The primary disadvantage of the previously reported adsorption studies is that they are generally applied at the laboratory scale without any pilot study or commercial-scale column filtration system. On top of the limitations of the adsorbents used, the bulk of the research work employed batch mode experiments with simulated mono-pollutant solutions, with just a handful using genuine wastewater. Most investigations on bio-waste adsorption focused on removing a single contaminant from actual dye-containing effluent. To meet the needs of wastewater treatment, more research should be conducted in multi-pollutant systems with real textile wastewater. Additionally, the review demonstrates certain inherent limits of recent developments in the use of activated carbon in terms of operational efficiency, overall costs, energy consumption, and the potential to form harmful by-products, even when these approaches work well against a specific pollutant. Although most bio-wastes had high elimination efficiency up to 99%, various and different parameters were used as indicators in the previous research works, which limits the potential for comparative studies. Finally, most of the previous research works on biomass-based adsorption were undertaken at a laboratory scale using simulated wastewater, and thus, the undertaking of cost analyses was restricted.

## 13. Conclusions and Recommendations

Bio-waste is the richest economically available source of carbon synthesis and is often transformed into activated carbon. From 2012 to 2021, bio-waste has emerged as a low cost, effective, and renewable source of activated carbon for the removal of MB. Low-cost bio-waste-derived adsorbents can be characterized and defined in terms of their initial costs, local availability, stability, eco-friendliness, transportation, applied treatment processes, recycling, lifespan concerns, regeneration potential, and pore volume after deactivation. In terms of the parameters that influence performance, the most critical characteristic affecting the adsorption of cationic dyes is the pH level; high pH values are necessary to achieve maximum dye uptake. Additionally, the initial dye concentration, temperature, adsorbent dose, type, and contact duration are the parameters that determine the dye-adsorption ability.

The processing methods employed in the adsorption studies include activation by steam, carbon dioxide, and chemical methods. Steam activation is the most cost-effective approach, whereas chemical activation produces the highest porosity and surface area. In terms of regeneration processes, the available desorption methods include thermal acid and nitric acid, sodium hydroxide, organic solvent, vacuum, and biological approaches. For a cost-effective system, the volume of adsorbent used, the simplicity of preparation or processing, green chemistry ideas, and the activation process used are the factors that can be considered. Additionally, catalyzer, ceramic, and fertilizer applications all show potential in the management of post-adsorption material.

In a nutshell, the adsorbent’s stability and affordability are other important characteristics that influence its applicability in terms of ensuring an efficient on-site treatment. Local availability, transportation, economic feasibility, potential for regeneration, and lifespan difficulties can also be investigated in future research works. Regeneration studies are also necessary to reduce process costs, recover adsorbed pollutants, and reduce waste generation.

## Figures and Tables

**Table 1:** Structural characteristics and adsorption capacity of adsorbent in relation to the efficiency of the elimination of MB within the 2008 to 2020 period.

No	Adsorbents	Surface Area (m^2^/g)	Diameter, ɸ (nm)	Q_max_ (mg/g)	Sources
1	Activated charcoal	4.445–2854	1.0–15.9	0.71–1030	[35,36,37,38,39,40,41]
2	Biochar	2.05–2054.49	2.29–20.57	2.06–1282.6	[42,43,44]
3	Modified activated carbon and modified biochar	4.02–1229	1.038–7.477	9.72–986.8	[45,46,47]
4	Carbon graphics and modifications	32–295.56	2–50	41.67–847	[45,47,48,49]
5	Porous Carbon	21–3496	0.74–5.45	8.96–1791	[50,51,52,53]
6	Carbon Nanotube	140–558.7	2.19–25	33.4–1189	[49,54,55,56,57,58,59]

**Table 2:** Benefits and drawbacks of various wastewater treatment technologies for MB’s removal.

Technologies	Benefits	Drawbacks	Reference
Advanced oxidation process	At normal atmospheric pressure and temperature, the dyes are degraded efficiently, and organic contaminants are transformed into carbon dioxide.	Significant operating and maintenance expenses; inflexibility	[69,73]
Chemical precipitation	Simple; low-cost; can manage high pollutant loads; is easy to use; has an integrated physio-chemical process; and results in a significant reduction in COD.	Contains a huge amount of chemicals and generates a lot of sludge	[82]
Ion exchange	Absence of sludge; requires less time; water of superior purity is generated; and an effective decolorization procedure is used. No adsorbent loss during regeneration	pH has a significant effect on performance; not suitable for all colors; costly in terms of recharging and the formation of significant amounts of sludge	[73,81]
Electrochemical	Chemicals are either unnecessary or are limited; the process is quick; suited to both insoluble and soluble dyes, with a lower COD.	High operating expenses; rising electricity prices; sludge formation; contamination from chlorinated organics and heavy metals as a result of indirect oxidation	[65,69]
Oxidation	Dyes are completely degraded, and the reaction time is minimal.	pH maintenance; catalyst required for optimal treatment; high cost	[69,83]
Ozonation	Disinfection that is quick and effective, as well as equipment installation that is simple; no volume growth in the gas phase	A relatively brief half-life; costly process; hazardous by-products and intermediates in manufacturing; strict pH control of effluent	[81,84]
Hydrogen peroxide	Oxidation causes water-insoluble colors to decolorate; reduction in COD; and non-toxic by-products of manufacturing	Increased reaction time; increased need for space; more costly	[65]
Fenton reagents	Removal of both soluble and insoluble dyes with effective decolorization	Sludge production	[63]
Sodium hypochloride	Cleavage of azo bonds develops and accelerates	Production of aromatic amines	[63]
Electrochemical destruction	The breakdown products are not dangerous.	Electricity is costly	[63]
Coagulation–Flocculation	A wide range of physiochemical approaches used for color elimination; the coagulating agent entirely removes dyes from remediated wastewater; it is effective and simple to operate, and as a result decolorization occurs completely.	Recycling high-priced chemicals is impractical; not suited for very water-soluble colors; generates colorful coagulated solid waste; produces hazardous sludge; raises TDS in treated wastewater; is not ecologically sustainable.	[65,82]
Ultrafiltration and Nanofiltration	Effective with all types of dyes	Extreme operational pressure, significant energy consumption, high price of membrane, limited lifespan, and concentrated production of sludge	[83,85,86]
Reverse osmosis	The most efficient decolorizing and desalting technology, with maximal salt removal, and high-quality water	Extreme pressure and operating costs, as well as membrane clogging, are involved on a frequent basis.	[83,86]
Biological techniques (aerobic and anaerobic)	Low-cost, environmentally friendly, and non-dangerous product; is fully mineralized.	Dye biodegradability is lower, extremely dependent on reaction circumstances, design and operation inflexibility, requires a vast land area, and the requires a longer period for decolorization	[69]
Adsorption technique	Highly efficient and easy; simple and adaptable to a wide variety of pollutants; excellent capacity to remove a wide variety of impurities; economical; adsorbents can be made from wastes; potential regeneration of the adsorbent	Adsorbents’ compositions influence their efficacy; their chemical modification is necessary to boost their adsorption capacity; certain adsorbents are highly expensive.	[83,86]

**Table 3:** Classification of various carbon compounds and their associated benefits and drawbacks.

Classifications	Adsorbents	Formation	Benefits	Drawbacks	Sources
Composition of carbon	Activated carbon	Carbonized and activated (e.g., lignite, coal, peat, wood)	Large and specific chemical functional groups; large surface area; large pore volume	Hygroscopicity; pore resistance; flammability; incomplete desorption; high permeability	[104]
	Biochar	Formed under moderate pyrolysis conditions in an inert environment	Abundant resources; highly efficient; affordable; low energy usage	Plug hole; flammability; hygroscopicity; gas release	[105]
	Carbon fiber, activated	A microfilament fiber	Hydrophobic and efficient	Expensive	[106]
	Graphene	2D graphene is made up of carbon sheets hexagonal that portion three extra carbon atoms’ sp2 hybridized orbitals	Superior electrical conductivity; a large amount of physical specific surface area; great mechanical strength	Synthesis is difficult and dangerous	[107]
	Carbon nanotubes	The cylindrical structure is composed of carbon atoms that have undergone sp2 hybridization.	Strong thermal stability; good electrical conductivity; wide surface area; inherent hydrophobicity	Serious aggregation	[108]
Materials containing oxygen	Zeolite	Zeolite is composed of an endless (3D) arrangement of TO4 tetrahedra in a crystalline aluminosilicate frame (T is Al or Si)	High adsorption capacity; huge surface area; tunable porosity; incombustibility; hydrothermal and chemical stability; good hydrophobicity	The synthetic technique is intricate, lengthy, and costly	[109]
	Frameworks of metal organic	Metal ions or coordination clusters containing organic ligands are created in a single-, two-, or three-dimensional manners.	Extremely large surface area; outstanding thermal stability; oxidizable porous structure; simplicity of functionalization	A large vacuum space; a weak dispersion force; an unsuitable environment for coordination; an inadequate number of active metal catalyst areas; expensive preparation costs	[110]
	Clay	Clay is a layered aluminosilicate mineral that contains water and is found in rocks and soils	Strong thermal stability; excessive heat resistance; great surface area; a special micro-porous medium; inexpensive cost	Because of its underdeveloped pore structure, clay’s adsorption affinity for carbon-based gases is restricted	[111]
	Silica gel	Silica gel is a three-dimensional tetrahedral inorganic substance with silicol groups on its surface	Low density; substantial porous surface area; multiple functional groupings; excellent mechanical, thermal, and chemical stabilities	Hygroscopicity	[112]
Organic polymers	Macroporous and hyper cross-linked polymers	Other known porous materials have a higher density than organic polymers made of light nonmetallic components such as C, H, O, N, and B	Large specific surface area; excellent porosity; low weight; excellent thermal stability, repeatability, and hydrophobicity	Complex synthesis	[113]

**Table 14:** Desorbing agents for various adsorbents.

Adsorbents	Desorbing Agents	Agent	References
Chemical sorbents	Alkali	NaOH	[295]
Bio-adsorbents	Acid	HCl, H_2_SO_4_, HNO_3_	[295]
Biomass (fungi, algae)	Complexing agents	EDTA	[295]

## Data Availability

Not applicable.
